# BET and Aurora Kinase A inhibitors synergize against *MYCN*-positive human glioblastoma cells

**DOI:** 10.1038/s41419-019-2120-1

**Published:** 2019-11-21

**Authors:** Matko Čančer, Lisa F. Drews, Johan Bengtsson, Sara Bolin, Gabriela Rosén, Bengt Westermark, Sven Nelander, Karin Forsberg-Nilsson, Lene Uhrbom, Holger Weishaupt, Fredrik J. Swartling

**Affiliations:** Department of Immunology, Genetics and Pathology, Science For Life Laboratory, Uppsala University, Uppsala, Sweden

**Keywords:** Targeted therapies, Preclinical research

## Abstract

Glioblastoma multiforme (GBM) is the most common primary malignant brain tumor in adults. Patients usually undergo surgery followed by aggressive radio- and chemotherapy with the alkylating agent temozolomide (TMZ). Still, median survival is only 12–15 months after diagnosis. Many human cancers including GBMs demonstrate addiction to MYC transcription factor signaling and can become susceptible to inhibition of MYC downstream genes. JQ1 is an effective inhibitor of BET Bromodomains, a class of epigenetic readers regulating expression of downstream MYC targets. Here, we show that BET inhibition decreases viability of patient-derived GBM cell lines. We propose a distinct expression signature of *MYCN*-elevated GBM cells that correlates with significant sensitivity to BET inhibition. In tumors showing JQ1 sensitivity, we found enrichment of pathways regulating cell cycle, DNA damage response and repair. As DNA repair leads to acquired chemoresistance to TMZ, JQ1 treatment in combination with TMZ synergistically inhibited proliferation of *MYCN*-elevated cells. Bioinformatic analyses further showed that the expression of *MYCN* correlates with Aurora Kinase A levels and Aurora Kinase inhibitors indeed showed synergistic efficacy in combination with BET inhibition. Collectively, our data suggest that BET inhibitors could potentiate the efficacy of either TMZ or Aurora Kinase inhibitors in GBM treatment.

## Introduction

GBM is characterized by considerably high rates of proliferation, invasion, and neovascularization, making this severe brain tumor particularly difficult to cure^1^. Standard course of treatment for GBM patients includes surgical resection as well as radio- and chemotherapy^2^. The alkylating agent temozolomide (TMZ) increases median patient survival^3^; however, only from 12 to 15 months. GBM occurs either as primary or secondary glioblastoma, with median onset age of 55 and 45 years, respectively^4^.

GBM can be further classified based on expression profiles into three molecularly defined subtypes^5,6^: proneural, classical, and mesenchymal. Alterations in *PDGFRA* and point mutations in *IDH1* are particularly prevalent among proneural GBM, classical GBM is characterized by amplifications of *EGFR*, while deletions in *NF1* are dominant in mesenchymal subtype. However, it has become clear that GBM subtype specification is presumably an enrichment in a particular signature and it is rather common to see more than one signature activation in patients’ biopsies^6^. This complexity of GBM tumor forms and subtype heterogeneity is likely a reason behind the fact that a selective and targeted therapy has still not been described, leaving patients with TMZ as the only option for GBM targeting.

*MYC* is an important oncogene that was first discovered from an avian retrovirus over 30 years ago (*v-myc*)^7^, while its cellular homolog, *MYC* has been later found overexpressed in many human cancers and described as a driving force of malignant transformation and uncontrolled proliferation^8^. A recently developed dominant-negative binding partner of MYC, termed OmoMYC, successfully inhibited MYC homo- and heterodimerization, thus preventing cell division and inducing mitotic crisis in GBM models^9^, demonstrating the oncogenic addiction of GBM cells to MYC signaling. Since GBM shows addiction to MYC signaling^9–12^, which is absent in the adult brain, MYC proteins are believed to be suitable therapeutic targets. A clinically available direct inhibitor of either MYC or its family member MYCN has not yet been developed. Inhibition can, however, be achieved through epigenetic silencing of *MYC* genes or by inhibiting signaling pathways downstream of the MYC transcription factor.

Regulation of the transcription of *MYC* genes can be mediated through bromodomain and extra terminal (BET)-containing epigenetic readers. BET proteins are a class of proteins that specifically recognize acetylated lysine residues on histones^13^, where the BET-containing protein BRD4 has been abundantly found at the promoter regions of *MYC* genes^14^. *MYC* transcription can be effectively and specifically targeted through BET inhibition, as it has been demonstrated in neuroblastoma, medulloblastoma, and glioblastoma^15–17^ using the small molecule inhibitor JQ1.

Here we present a rationale for indirect epigenetic and downstream inhibition of MYC signaling together with TMZ as a potential therapeutic strategy for a subset of proneural GBM that presents a specific sensitivity expression signature.

## Results

### BET inhibition results in differential response of human glioblastoma cell cultures (HGCCs)

**Fig. 1:**
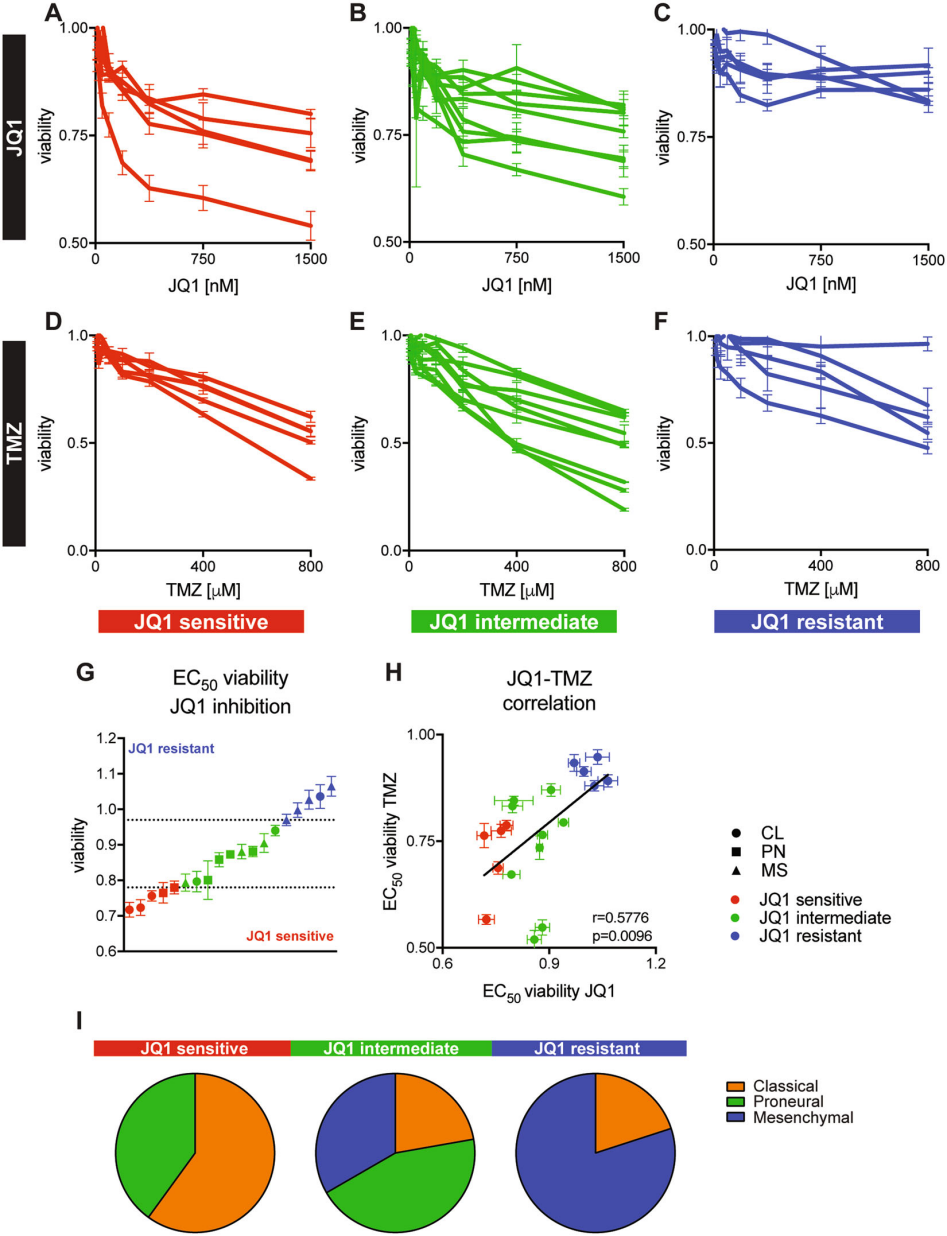
In vitro screening of 18 patient-derived GBM cell cultures for response to JQ1 and TMZ. JQ1 dose response curves were used to define JQ1-sensitive (**a**), JQ1-semi-sensitive (**b**) and JQ1-resistant class (**c**) of GBM cell lines. Unlike JQ1, TMZ successfully inhibited proliferation and viability of GBM cell lines used in this screen (**d**–**f**). When exposed to 500 nM JQ1 (EC_50_ concentration), 18 patient-derived GBM cell cultures exhibit differential viability response again showing three JQ1 response classes (**g**). Viability of assayed 18 GBM cell lines upon 500 nM JQ1 (EC_50_) inhibition significantly correlates (Pearson correlation) with viability upon 400μM TMZ inhibition (**h**). Classical and proneural GBM subtype dominated in the JQ1-sensitive class, while the JQ1-resistant class was represented by mainly mesenchymal GBM cell lines (**i**). CL classical, PN proneural, MS mesenchymal subtype.

Many human cancers including GBMs demonstrate oncogenic addiction to MYC signaling^9,10,18^. To find out whether this is true in our experimental model, we performed a JQ1 inhibition screen on 18 patient-derived GBM cell cultures^19^ representing different GBM molecular subtypes (Table 1). Based on their response to inhibition, we were able to stratify GBM cell cultures into JQ1-sensitive (Fig. 1a), JQ1-intermediate (Fig. 1b) and JQ1-resistant groups (Fig. 1c). While JQ1-sensitive and JQ1-intermediate groups demonstrated dose-dependent decrease in cell viability up to 500 nM, JQ1 had very little effect on reducing cell viability in the resistant group (Fig. 1a–c), indicating that at concentration higher than that, the binding of JQ1 to BET proteins most likely reached saturation and the excess of drug will not have any effect on the inhibition. When we exposed the cells to TMZ, which is the standard chemotherapeutic drug used for GBM patients in the clinic, we observed a dose-dependent decrease in cell viability across 17 out of 18 GBM cell cultures (Fig. 1d–f). JQ1 inhibition showed a differential suppression of viability that stratified the cell cultures into three groups, while TMZ treatment reduced viability in all cell cultures to the same level. A summary of all EC_50_ concentrations for all 18 GBM cell lines for JQ1 and TMZ is presented in Table 1.

**Table 1:** Glioblastoma patient-derived cell line models, age at diagnosis, survival, EC50 of JQ1 and TMZ.

Cell line	Age	Survival (days)	Subtype	JQ1 EC50 (nM)	EC50 TMZ (µM)
U3005MG	65	26	PN	128.4	418.7
U3009MG	60	174	CL	122.4	481.5
U3013MGa^a^	78	122	PN	537.7	381.7
U3013MGb^a^	78	122	PN	386.7	579.2
U3020MG	68	160	MS	118.3	462.8
U3021MG	50	387	PN	970.2	301.8
U3024MG	73	170	MS	605.9	192.1
U3027MG	44	766	MS	386	343.1
U3028MG	72	496	CL	331.7	353
U3037MG	60	333	MS	307.4	471.2
U3042MG	67	202	CL	132.8	375.3
U3054MG	60	611	MS	1124	589.8
U3056MG	68	110	CL	94.42	297.2
U3073MG	71	481	MS	233.7	343.5
U3082MG	70	314	PN	786	193.3
U3084MG	72	444	CL	7.855	157.6
U3086MG	72	444	CL	171,7	663.5
U3110MG	58	243	MS	433.4	379.9
U3117MG	57	793	PN	486.5	456

EC50 values were computed using parameters described in Table S1. Patient age, survival and subgroup data obtained from ref. ^19^

*CL* classical, *PN* proneural, *MS* mesenchymal

^a^Subclones derived from the same cell line

Since we had seen very little effect of JQ1 beyond 500 nM, we exposed all 18 GBM cell cultures to 500 nM JQ1 for 72 h and measured viability. Similar to the dose–response experiments, GBM cells showed differential viability upon inhibition. Based on 25 and 75 percentiles as cut-offs, GBM cells were clustered as JQ1-sensitive when viability was below 78%, intermediate (78–98%), and resistant when viability was above 98% (Fig. 1g). When we analyzed sensitivity to JQ1 based on the area under the curve (AUC) of JQ1 dose–response curve (Fig. 1a–c), we found that most of the cell lines kept their sensitivity subgroup, with three cell lines shifting from being sensitive to intermediate, three cell lines from intermediate to sensitive, one cell line from intermediate to resistant and finally one cell line from being resistant to intermediate (Fig. S1a). Moreover, the viability of cell lines at 500 nM of JQ1 correlated with AUC values (*r* = 0.5575, *p* = 0.0131). Cells that were more sensitive to JQ1 inhibition at 500 nM concentration were also more susceptible to 400 μM TMZ treatment, as shown in Fig. 1h.

Many anticancer therapeutics rely on cell proliferation to kill cancer cells^20^ and therefore highly proliferative GBM cells may be more susceptible to JQ1 and TMZ inhibition. To investigate if this was true in this GBM subset, we analyzed sensitivity parameters (viability at common EC_50_ values and AUC for JQ1 and TMZ) in respect to doubling time (Fig. S1b–e). We found no correlation between drug response and doubling time in 18 GBM cell lines suggesting that the drug response is target specific and not likely to be attributed to a differential proliferation rate.

Interestingly, when we looked at the GBM subtype stratification within the JQ1 response groups, we could see a higher proportion of classical and proneural subtypes in the JQ1-sensitive group, while the JQ1-resistant group displayed an enrichment of cultures with a mesenchymal subtype (Fig. 1i).

### JQ1-sensitive GBM cells demonstrate a distinct expression signature

**Fig. 2:**
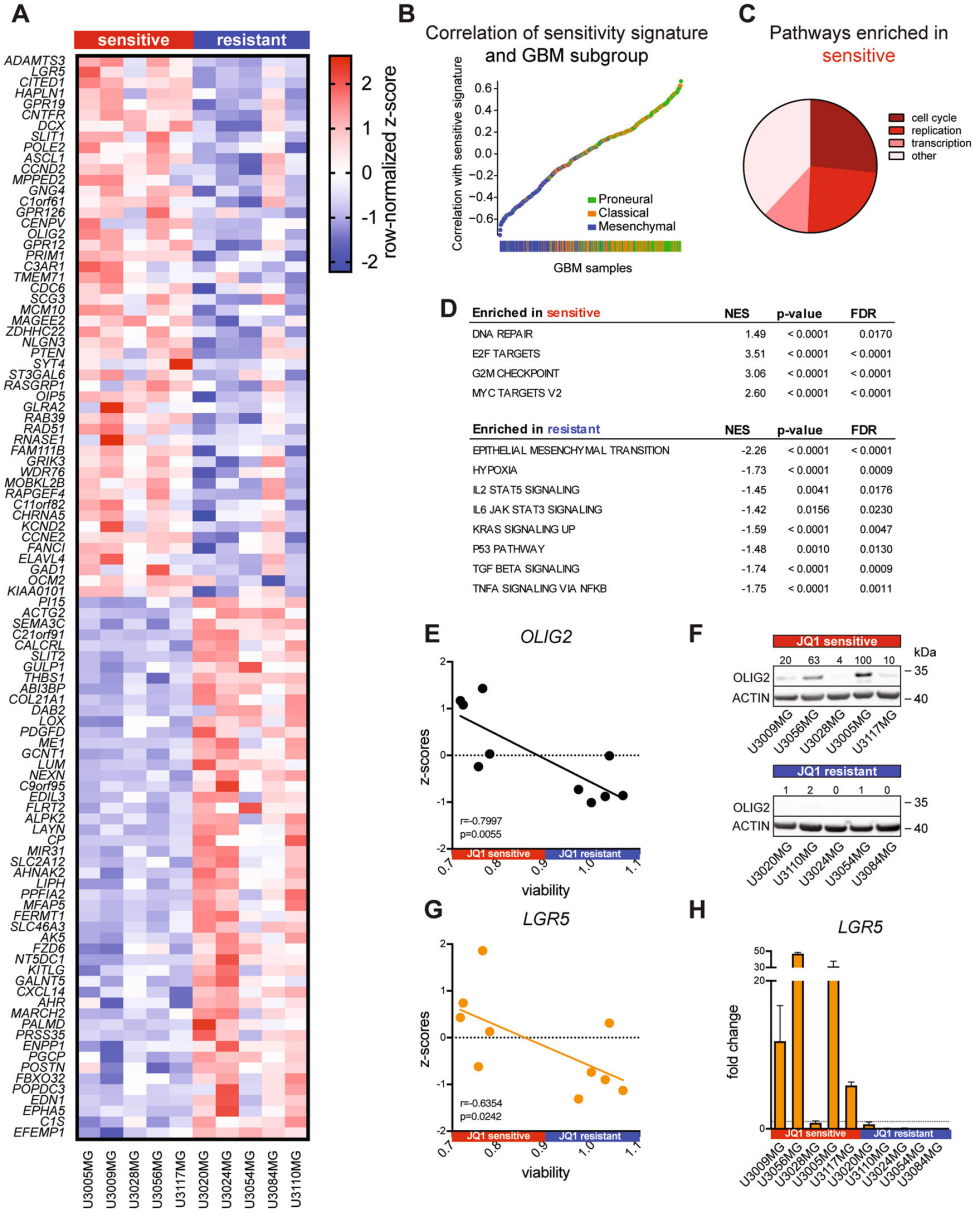
Expression profiling and comparison of JQ1-sensitive and resistant class of GBM cell lines. JQ1-sensitive cell demonstrated a distinct expression signature characterized by overexpression of *LGR5*, *PTEN* and *OLIG2* (**a**, see also Table S2). Distinct sensitivity signature is highly enriched among TCGA patient samples classified as proneural GBM (**b**). Pathway analysis showed enrichment of genes regulating cell cycle, DNA replication and transcription (**c**). JQ1-sensitive cells were enriched in genes regulated by *MYC* and involved in DNA repair, while JQ1-resistant cells were enriched in genes regulating EMT, hypoxia, and p53, TGFβ and TNFα signaling (**d**). *OLIG2* expression correlates (Pearson correlation) with the sensitivity to JQ1 inhibition (**e**) and high expression of *OLIG2* was confirmed by immunoblotting (**f**). Values above the blot represent relative quantification normalized to ACTIN and U3005MG. Similar to *OLIG2*, the expression of *LGR5* correlates (Pearson correlation) with the JQ1 sensitivity (**g**) and the expression of *LGR5* was confirmed by real-time quantitative PCR (**h**).

Different JQ1 response groups clearly showed differential enrichment of GBM subtypes, which prompted us to further investigate the difference in their expression profiles. Interestingly, when we compared expression profiles of JQ1-sensitive cells with JQ1-resistant ones using data available through www.hgcc.se and published in^19^, we identified a distinct expression signature characterizing those two classes (Fig. 2a). These genes represent the top 50 upregulated genes in JQ1-sensitive and JQ1-resistant cells, respectively. We then performed enrichment analysis on TCGA GBM samples, where we found a strong correlation of the sensitivity signature with the proneural subtype (Fig. 2b). Although not significant, proneural GBM patients enriched in JQ1-sensitive signature (79 samples positively correlated, median survival 316 days, Fig. S2a) displayed a longer median survival compared to proneural subtype samples with the resistant signature (seven samples negatively correlated, median survival 183 days, Fig. S2a). Proneural GBM subtype is often characterized by IDH1 mutations that significantly correlate with better prognosis and survival^5,21^. We next analyzed IDH1 mutation status in respect to JQ1 sensitivity signature, where we found no correlation (Fig. S2b). When we performed pathway enrichment, we found the JQ1-sensitive class to be enriched in signaling pathways associated with cell cycle, DNA replication and transcription (Fig. 2c). We then compared expression signatures of JQ1-sensitive and JQ1-resistant cell cultures. Interestingly, we found enrichment of genes associated with DNA repair and MYC target genes in the sensitive class (Fig. 2d, top), while the resistant class of GBM cultures was enriched in genes associated with EMT, hypoxia, JAK-STAT, TGFβ, and TNFα signaling (Fig. 2d, bottom). Among the top 20 genes upregulated in the JQ1-sensitive class of GBM cells were *OLIG2* and *LGR5* (Fig. 2e–h), where we found a strong correlation between the sensitivity to JQ1 and the expression of respective genes (Fig. 2e, g). The OLIG2 transcription factor is typically expressed in proneural GBM^5^ and is promoting stem cell characteristics^22,23^ as well as conferring resistance to genotoxic substances by modulating the p53 pathway^24^. LGR5 is an epithelial stem cell marker that is involved in promoting tumorigenicity and invasion of glioma stem cells^25^.

### JQ1 regulates cell cycle, DNA damage response and cell death in JQ1-sensitive GBM cells

**Fig. 3:**
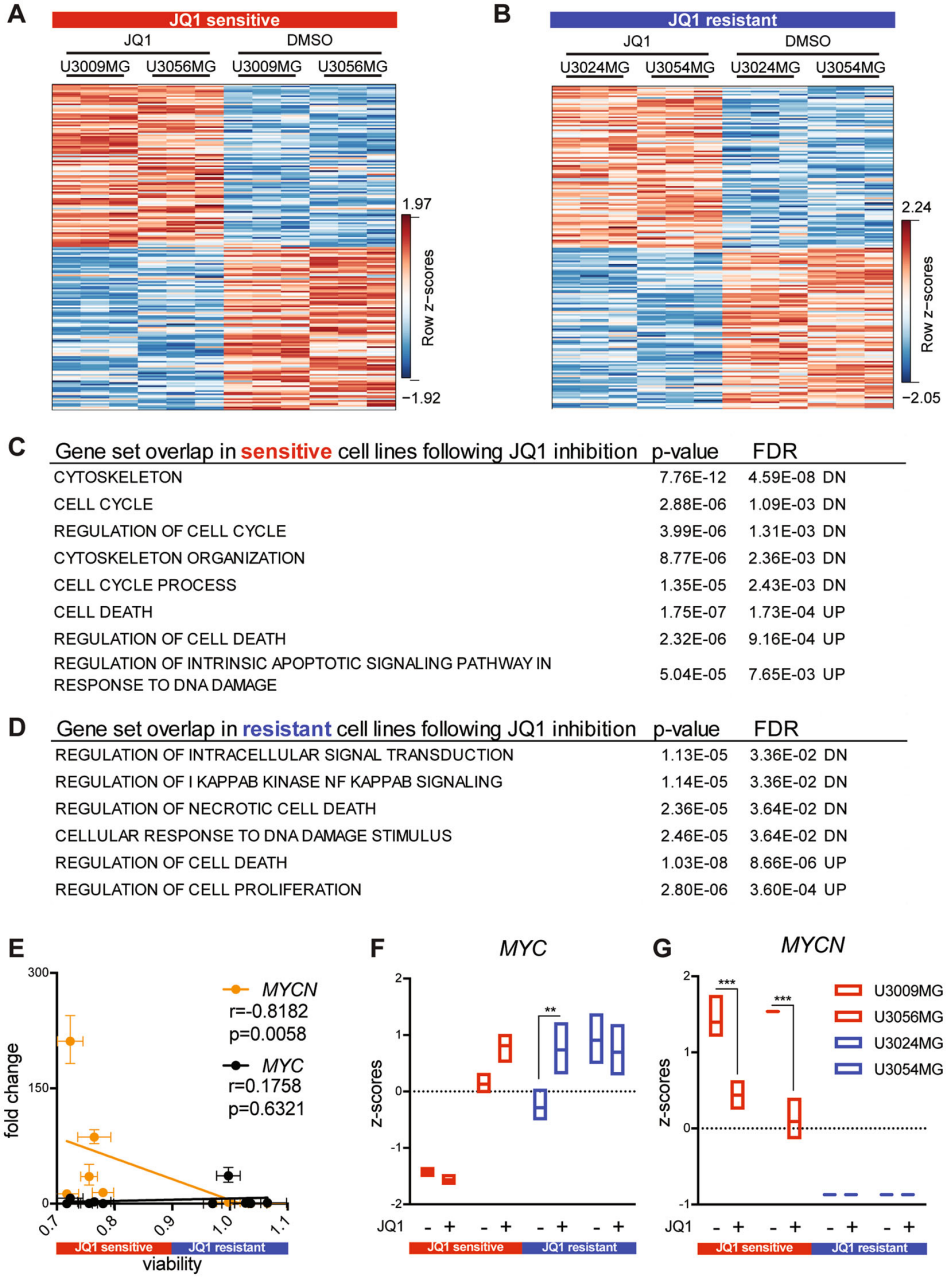
Expression profiling of selected JQ1-sensitive and JQ1-resistant GBM cell lines following JQ1 treatment. **a**, **b** Top 100 differentially expressed genes in JQ1-sensitive and JQ1-resistant GBM cell lines upon 6 h JQ1 inhibition. Gene names (rows) can be found in Tables S3–S6. **c** Gene set overlap showing direct suppression of genes regulating cell cycle and cytoskeleton, and upregulation of cell death mechanisms in JQ1-sensitive cells. **d** Gene set overlap following JQ1 inhibition of JQ1-resistant cells showing suppression of genes regulating intracellular signal transduction, necrosis and response to DNA damage, and upregulation of genes regulating cell proliferation. **e** *MYCN* significantly correlates (Spearman correlation) with sensitivity to JQ1, where high *MYCN* expression confers sensitivity in GBM cell lines. On the other hand, *MYC* expression showed no correlation (Spearman correlation) with the sensitivity to JQ1 inhibition. **f** 6 h JQ1 inhibition (500 nM) failed to significantly decrease expression of *MYC*, with one resistant GBM cell line upregulating its expression. **g** Following 6 h JQ1 treatment (500 nM), the sensitive cell lines dramatically decreased *MYCN* expression, while the resistant cells showed absence of *MYCN* in both treated and untreated conditions.

To investigate direct targets of JQ1 inhibition, we exposed two JQ1-sensitive lines U3009MG and U3056MG, and two JQ1-resistant lines U3024MG and U3054MG to JQ1 for 6 h and applied RNA sequencing to determine changes in gene expression. We identified a number of differentially expressed genes in both JQ1-sensitive and JQ1-resistant cultures when comparing JQ1 inhibition with DMSO treatment (Fig. 3a, b, Tables S3–S5). A detailed analysis of gene set overlaps revealed suppression of genes regulating cell cycle and activation of cell death mechanisms in JQ1-sensitive cells upon JQ1 inhibition (Fig. 3c). In contrast, the JQ1-resistant class demonstrated suppression of NFκB pathway and activation of pathways regulating cell proliferation (Fig. 3d). Intriguingly, in both the JQ1-sensitive and the JQ1-resistant class we identified cell death regulation as a specific JQ1 target. Within cell death regulation gene set, only four genes were upregulated in both JQ1-sensitive and resistant cells—*DNAJC3*, *SIRT1*, *BTG1*, and *STK17B*. Other genes involved in regulation of cell death that were upregulated in JQ1-resistant cells following 500 nM JQ1 inhibition were predominantly involved in anti-proliferative pathways (*BTG1*, *DUSP6* and *RPS6KA1*), cellular stress response (*HERPUD1* and *HSP1B*) and protection from apoptosis (*JUND*). By contrast, JQ1-sensitive GBM cells upregulated genes that *e.g*. promote activation of apoptotic pathways (*BCL2L11*, *BCL2L12*, and *PAK2*), suppress proliferation (*GADD45B*) or induce protein degradation (*USP47*). As sensitive and resistant cells exhibit a differential response to JQ1 inhibition, it is likely that JQ1 promotes cell death in sensitive cells, while in JQ1-resistant cells JQ1 inhibition suppresses cell death signals. Since JQ1 regulates cell cycle, DNA damage response and cell death, it is likely that correlation between JQ1 and TMZ sensitivity (Fig. 1h) arises because of the overlap in cellular responses upon their inhibition.

Following unbiased gene set overlap, we wanted to further investigate discrete changes in the typical JQ1 targets, *MYC* and *MYCN*^17^. We found a strong correlation between *MYCN* expression and JQ1 sensitivity (Fig. 3e), where high *MYCN* levels conferred JQ1 sensitivity in GBM cell lines and vice versa. However, *MYC* expression did not show any correlation with the sensitivity towards JQ1. We next investigated changes in *MYC* and *MYCN* expression following a short 6 h treatment with 500 nM JQ1. While this inhibition failed to suppress *MYC* expression in both sensitive and resistant cell lines (Fig. 3f), *MYCN* expression was significantly suppressed following JQ1 inhibition in the sensitive GBM cells (Fig. 3g). On the other hand, JQ1-resistant cells showed no *MYCN* expression in any of the conditions (Fig. 3g). Collectively, these data imply that the JQ1 sensitivity is likely to be coupled to the *MYCN* expression in human GBM, rather than *MYC*.

### JQ1 sensitizes glioblastoma cells to temozolomide

**Fig. 4:**
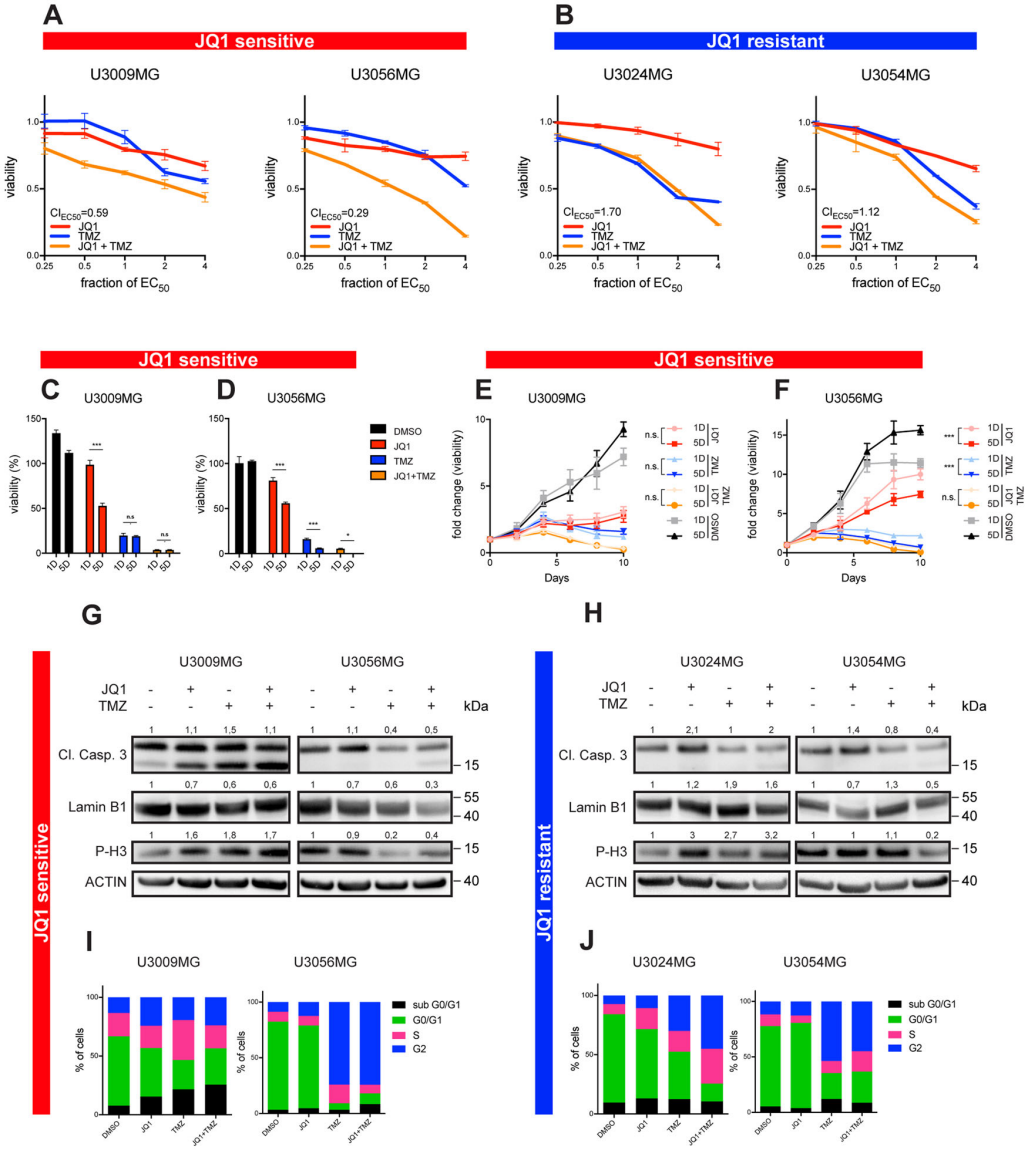
Combined JQ1 and TMZ inhibition of patient-derived GBM cell lines. JQ1 and TMZ demonstrated a strong synergism in JQ1-sensitive cells at the respective EC_50_ concentrations (**a**), while the two drugs showed antagonistic and mildly additive effect on respective JQ1-resistant GBM cell lines (**b**). Five-time treatment of U3009MG over a period of 10 days failed to increase efficacy of combined JQ1 and TMZ treatment compared to a single dose of inhibitors (**c**, **e**), while U3056MG cells showed less than 1% viability after five doses of JQ1 and TMZ (**d**, **f**). While JQ1-sensitive cells demonstrated increase in Cleaved Caspase 3, decrease in Lamin B1 and loss of phosphorylated histone H3 after JQ1, TMZ or combined inhibition (**g**), JQ1-resistant cells were only partial phospo-H3 reduction following TMZ inhibition (**h**). Similarly, JQ1-sensitive cells showed increase in subG_1_/G_0_ and G_2_ population following single agent or combined inhibition compared to control (**i**), while in JQ1-resistant population, the effect of combined JQ1 and TMZ was less prominent (**j**). CI = combination index (CI < 0.8: synergism; 0.8 < CI < 1.2: addition; CI > 1.2: antagonism). EC_50_ (JQ1) = 500 nM, EC_50_ (TMZ) = 400μM. See also Tables S7 and S8 for detailed statistical analysis related to (**e**, **f**). Immunoblotting was normalized to loading control (ACTIN) and untreated sample (DMSO control).

The JQ1-sensitive class of GBM cells was evidently enriched in DNA repair as well as demonstrating upregulation of genes associated with stemness, chemoresistance and cell cycle regulation. Therefore, we stipulated that by combining JQ1 with the alkylating drug TMZ, we could increase efficacy of single drug treatment in JQ1-sensitive GBM cells, but not in JQ1-resistant GBM cells. We next evaluated if there was a possible synergism between JQ1 and TMZ, and indeed demonstrated a strong synergistic effect in JQ1-sensitive lines U3009MG (CI_EC50_ = 0.59) and U3056MG (CI_EC50_ = 0.29) (Fig. 4a). This finding is in line with our data shown in Fig. 3c, where BET inhibition upregulated genes that promote apoptosis, inhibit protein synthesis and suppress proliferation, rendering those cells more susceptible to genotoxic effects of TMZ. As expected, we showed that JQ1 and TMZ combination is antagonistic to mildly additive in JQ1-resistant cells U3024MG and U3054MG (CI_EC50_ = 1.70 and CI_EC50_ = 1.12, respectively) (Fig. 4b), which is not surprising given the anti-apoptotic nature of JQ1 inhibition. To model continuous exposure to inhibitors and thus mimic the clinically relevant course of treatment, we exposed JQ1-sensitive cells to one dose or five doses of JQ1 (500 nM), TMZ (400 μM) or both agents over a period of 10 days. Despite the putative GBM heterogeneity that is conserved in cellular models^19,26^, we observed a nearly complete (Fig. 4c, e) and complete (Fig. 4d, f) reduction in viability of JQ1-sensitive cells U3009MG and U3056MG, respectively.

To investigate mechanisms behind the differential response to single and combined inhibition, we performed immunoblotting and cell cycle analysis. The JQ1-sensitive cell line U3009MG demonstrated significant increase in Cleaved Caspase 3 (Fig. 4g, left), while U3056MG showed reduction in Lamin B1 and phosphorylated histone H3 (Fig. 4g, right), suggesting two different mechanisms—apoptosis and senescence/cell cycle arrest, as a result of the same mode of inhibition. In line with immunoblotting data, a cell cycle analysis demonstrated increased apoptosis for U3009MG and cell cycle arrest for U3056MG (Fig. 4i). Reflecting that viability data showed little effects upon combined JQ1 and TMZ inhibition, JQ1-resistant cell lines U3024MG and U3054MG demonstrated no obvious change in apoptosis and senescence, but showed a subtle decrease in phosphorylated histone H3 indicating cell cycle arrest (Fig. 4h). The decrease in phosphorylated H3 prompted us to further investigate the proportion of cycling cells where we found that both JQ1-resistant cell lines showed a moderate arrest in S/G2 phase both upon TMZ or combined JQ1 and TMZ inhibition (Fig. 4j), confirming our previous data showing very little effect of JQ1 inhibition on JQ1-resistant cells.

### Aurora Kinase A inhibition synergizes with epigenetic *MYCN* expression inhibition

**Fig. 5:**
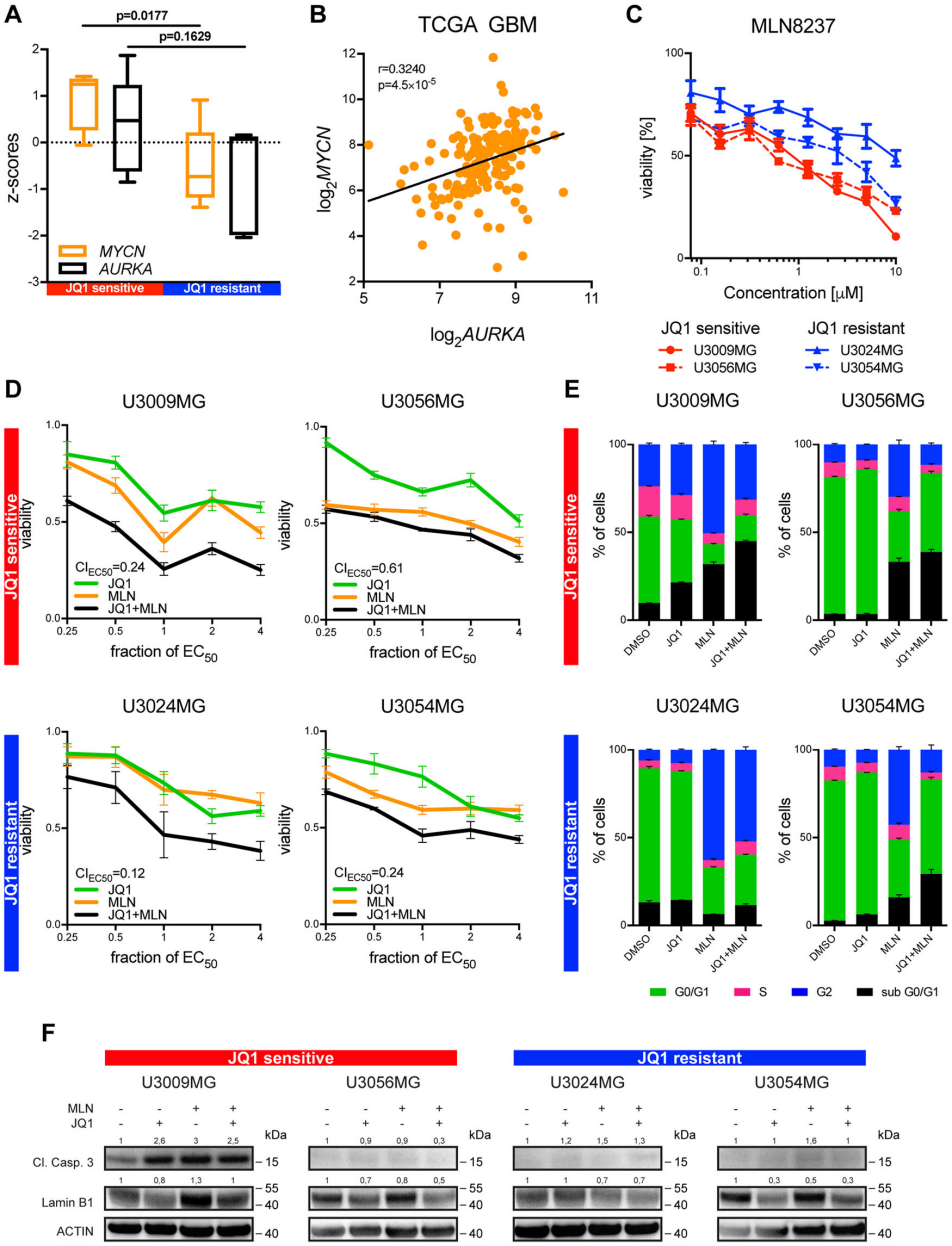
BET inhibition synergizes with Aurora A inhibition and promotes cell death in *MYCN* expressing GBM cells. JQ1-sensitive GBM cells showed significantly higher *MYCN* expression compared to JQ1-resistant cells, with a similar trend observed in the expression of *AURKA* (**a**, data downloaded from www.hgcc.se). In a large GBM patient cohort (TCGA) the expression of *AURKA* positively correlates with *MYCN* expression (Pearson correlation, n = 153) (**b**). JQ1-sensitive cells demonstrated significantly (see Table S9) stronger reduction in viability following Aurora A inhibition using small molecule inhibitor MLN8237 compared do JQ1-resistant cells (**c**). Combined BET and Aurora A inhibition was synergistic in both JQ1-sensitive and resistant GBM cell lines (**d**). While in JQ1-sensitive cells the dual inhibition resulted in cell cycle arrest and apoptosis, JQ1-resistant cells were predominantly arrested in either G0/G1 or G2 phase (**e**, see also Supplementary Table S10 for multiple comparison-adjusted p-values). While cellular senescence was induced in both JQ1-sensitive and resistant GBM cells as demonstrated by a decrease in Lamin B1 following JQ1 or dual inhibition, only one of the JQ1-sensitive GBM cell lines demonstrated a significant upregulation of Cleaved Caspase 3, indicating an induction of apoptosis (**f**). EC_50_ (JQ1) = 500 nM, EC_50_ (MLN8237) = 750 nM. CI = combination index (CI < 0.8: synergism; 0.8 < CI < 1.2: addition; CI > 1.2: antagonism). Immunoblotting was normalized to loading control (ACTIN) and untreated sample (DMSO control).

JQ1-sensitive cells demonstrated a clear difference in *MYCN* expression as compared to JQ1-resistant cells (Fig. 3g) and moreover showed a significant suppression of *MYCN* following BET inhibition. MYCN transcriptionally activates the expression of *AURKA*, an important kinase involved in the formation of mitotic spindles and cell cycle progression through mitosis^27^. Aurora Kinase A (Aurora A, encoded by *AURKA* gene) is required for the growth of *MYCN* amplified neuroblastoma cells, while in neuroblastoma cells with a low *MYCN* expression, levels of *AURKA* are dispensable^28^. In JQ1-sensitive GBM cells with a high expression of *MYCN* we found a trend towards upregulation of *AURKA*, while in GBM cells with a significantly lower *MYCN* expression *AURKA* expression was substantially lower (Fig. 5a). In a large number of GBM patients, elevated *AURKA* expression significantly correlated with increased *MYCN* expression (Fig. 5b). In line with that, Aurora A inhibition using a small molecule inhibitor MLN8237 (Alisertib) resulted in a significantly greater reduction in viability of GBM cells with a higher *AURKA* expression (JQ1-sensitive cells), as compared to cells with a lower *AURKA* expression (JQ1-resistant cells, Fig. 5c). As BET inhibition was shown to be synergistic with Aurora A inhibition in neuroblastoma cells^29^, we hypothesized that we could potentiate the effects of JQ1 on GBM cells by combining it with MLN8237. Similar to Felgenhauer et al.^29^ where they found synergism between BET and Aurora A inhibitors irrespective of *MYCN* status of neuroblastoma cells, we could demonstrate a strong synergism in all four tested GBM cell lines, irrespective of whether they had high (JQ1-sensitive) or low (JQ1-resistant) *MYCN* expression (Fig. 5d). However, while JQ1-sensitive GBM showed both cell cycle arrest and an increase in apoptosis after mono- and combination treatment (Fig. 5e, f), JQ1-resistant cells did demonstrate cell cycle arrest but only a modest upregulation of apoptosis and no apparent cell death (Fig. 5e, f). All four GBM cell lines showed, however, a decrease in Lamin B1, proposing an induction of cellular senescence following JQ1 or JQ1 + MLN8237 treatment. Collectively, our data suggest that BET inhibition may be combined with Aurora Kinase A inhibition to increase the efficacy of monotherapy, especially in cases where BET inhibition lacks a substantial effect on tumor cells.

## Discussion

Glioblastoma multiforme (GBM) is a highly heterogeneous disease^5^ with a particularly dismal prognosis and poor survival as a result of aggressive and invasive tumor growth^1^. Despite advances in understanding how GBM develops, there are still only a very limited palette of treatment options that include surgical resection, radio- and chemotherapy with TMZ^3^, bevacizumab^30^ or tumor-treating fields^31^.

MYC genes are upregulated or amplified in a vast variety of human cancers including GBM^8,10,32^, and suppression of MYC signaling has proven successful in cellular and mouse models of various human tumors^9,14–17,33^. We previously showed how efficient BET inhibitors, like JQ1, are regulating downstream transcriptional output of MYC and MYCN proteins in childhood brain tumors^34^. When similarly comparing expression profiles of JQ1-sensitive and JQ1-resistant patient-derived GBM cell cultures, we observed a distinct, clinically relevant signature defining a sensitive and a resistant class. Interestingly, JQ1-sensitive cells demonstrated enrichment in DNA repair mechanisms and should thus display a better response to alkylating agents. However, DNA repair is required for the TMZ mechanism of action where cytotoxic O^6^-methylguanine adducts induce double strand breaks through functional mismatch repair mechanism^35^. On the other hand, O^6^-methylguanine methyltransferase (MGMT) or base excision repair machinery can efficiently remove affected DNA adducts before causing harm to cells and thus give rise to chemoresistance in GBM cells^35,36^. As DNA repair can introduce novel mutations often leading to more aggressive growth and chemoresistance^35^, targeting DNA-repair mechanisms requires a precise and context-dependent planning as it might interact with TMZ mechanism of action. Collectively, our strategy to combine the cytotoxic effect of TMZ with BET inhibition modulates DNA damage response to overcome TMZ resistance as a consequence of active DNA repair mechanisms. However, future therapies that are based on inhibition of *MYCN* signaling should be carefully planned, as we demonstrated protective effects of JQ1 against genotoxic effects of TMZ. One plausible strategy is to combine BET inhibition with Aurora Kinase A inhibitors, that not only shows a promising anti-tumoral effect in JQ1-sensitive GBM cells, but also renders JQ1-resistant cells sensitive to the inhibition.

It is well known that cancer progression relies on unlimited and sustained proliferative capacity, and replicative immortality^37^. Hence, targeting components of signaling pathways involved in DNA replication and cell cycle progression represents an important rationale when designing novel therapeutic strategies in the battle against cancer. MYC as a transcription factor modulates many cellular processes involved in regulating cell cycle and CDKs were one of the first genes identified as MYC targets^38–41^. The precise regulation of CDKs and cyclins is fundamental to homeostasis and dysregulation inevitably leads to neoplastic growth and ultimately to malignant transformation^42,43^. Intriguingly, in the JQ1-sensitive class of GBM cells we found a strong enrichment of genes and pathways regulating DNA replication and transcription, and cell cycle. As we demonstrated, JQ1 alone facilitated accumulation of cells in subG_1_/G_0_ phase of cell cycle thus suggesting that, in addition to chemosensitizing effects, BET inhibition suppresses viability via cell cycle modulation.

Targeting glioma stem cells is one of the major challenges to overcome when it comes to tumors that are refractory to chemotherapy. Current therapeutic strategies employed against GBM vastly depend on killing rapidly dividing cells that are susceptible to ionizing radiation as well as cytotoxic effect of alkylating agents. However, a small fraction of slow cycling cells with stem-like properties is thought to be responsible for GBM recurrence and resistance to TMZ^44^. These cells reside in perivascular niches with readily available growth factors and matrix proteins that promote stem-like cell characteristics^44^. Among the top 20 most upregulated genes in the JQ1-sensitive class of GBM cells, we identified *OLIG2* and *LGR5*. *OLIG2* is known marker of proneural GBM subtype^5^ and ectopic expression of *OLIG2* together with other neurodevelopmental transcription factors can induce pluripotency in differentiated GBM cells^45^. Moreover, the deletion of *OLIG2* results in a phenotypic change from an oligodendrocyte-precursor signature towards a more differentiated expression pattern^23^, all suggesting that *OLIG2* plays an important role in glioma stem cell maintenance. Although explored to a lesser extent in glioma, *LGR5* expression promotes stem-like properties of cervical cancer^46^, promotes malignancy and modulates drug response of glioma stem-like cells^25^. Since JQ1-sensitive cells express high levels of both *OLIG2* and *LGR5*, we anticipate that BET inhibition specifically targets the proneural glioma stem cell niche and makes these cells even more susceptible to genotoxic effects of TMZ.

We provide here a rationale for combining the alkylating drug TMZ with epigenetic inhibitor JQ1 as a combination therapy against GBM. The major obstacle with the current treatment regimen, which involves TMZ and radiotherapy, is a refractory disease as a consequence of acquired chemo- and radioresistance. By modulating a number of target genes involved in DNA damage response and repair, whose expression is mediated through BET proteins bound in a superenhancer complex, we could render GBM cells sensitive to alkylating agents. As finally shown and irrespective of TMZ treatment, a combination of BET and Aurora A inhibition is effective in both JQ1-sensitive and JQ1-resistant GBM cells and could be used as a promising combination therapy for this devastating disease. Many prospective drugs in clinical trials have failed to provide a survival benefit for GBM patients because of blood-brain-barrier (BBB) that is in many cases impermeable to various cytotoxic drugs^47^. However, MLN8237 has a capacity to cross BBB^48^, making it even more an interesting candidate for future novel therapeutic strategy in combating GBM, either alone or in combination with radiation or other MYC-targeting drugs.

## Materials and methods

### Cell cultures, chemicals, and inhibitors

Human glioblastoma cell lines were obtained from Human Glioblastoma Cell Culture (HGCC) Resource and cultured as described^19^. All cell lines have been validated by STR profiling with HGCC consortium. JQ1 was obtained from James Bradner Laboratory, Harvard. Temozolomide (TMZ, cat# S1237) and MLN8237 (cat# S1133) were purchased from Selleckchem. Dimethyl-sulfoxide (DMSO, cat# 471267) was purchased from Sigma.

### Dose response and combination experiments

To analyze cell viability upon exposure to JQ1, MLN8237 or TMZ, 10 000 cells were seeded in three technical replicates, in Laminin (cat# L2020, Sigma) coated 96-well polystyrene plates. Inhibitors were provided at the beginning and every 24 h in 2-fold serial dilution ranging from 11.2 nM to 1,5 μM for JQ1, 39 nM to 10 μM for MLN8237 and 6.25 to 800 μM for TMZ. When calculating EC_50_ for JQ1 and TMZ, minimum response was taken as the viability at the lowest concentration and the maximum response was taken as the viability at the maximum concentration. Detailed analysis parameters are provided in the Table S1. To define JQ1 sensitivity subgroups, cells were exposed to 500 nM JQ1 (from now on called common JQ1 EC_50_) and the viability was measured. JQ1-sensitive cells we defined as cell lines with viability between minimum and 25 percentile, JQ1-intermediate as cell lines having viability between 25 and 75 percentile, and JQ1-resistant cells as cell lines with viability between 75 percentile and maximum. Similar analysis was performed in order to stratify cell lines into sensitivity groups based on area under the curve (AUC) of JQ1 dose–response curves. JQ1-sensitive cells were therefore defined as those having AUC between minimum and 25 percentile, JQ1-intermediate having AUC between 25 and 75 percentiles, while JQ1-resistant cells presented AUC from 75 percentile to maximum. To measure efficacy of combined JQ1 and TMZ inhibition, the two most JQ1-sensitive (U3009MG and U3056MG) and JQ1-resistant (U3024MG and U3054MG) cell lines were chosen. 10 000 cells were exposed to 125nM–2μM (2-fold range 0.25–4 × EC_50_) of JQ1, 100 μM–1.6 mM (2-fold range 0.25-4 × EC_50_) of TMZ, or both (2-fold range of both drugs, each drug having partial concentration range 0.25-4 × EC_50_). To measure efficacy of combined JQ1 and MLN8237 inhibition, the two most JQ1-sensitive (U3009MG and U3056MG) and JQ1-resistant (U3024MG and U3054MG) cell lines were chosen. 10,000 cells were exposed to 125 nM–2 μM (0.25–4 × EC_50_) of JQ1, 187.5 nM–3 μM (0.25–4 × EC_50_) of MLN8237, or both (2-fold range of both drugs, each drug having partial concentration range 0.25–4 × EC_50_). Cell viability was measured after 72 h by Resazurin assay and normalized against DMSO treated cells. Combination indices were computed using CompuSyn as described^49,50^. Combination index (CI) with a value of less than 0.8 indicated a synergy, between 0.8 and 1.2 indicated addition, and CI > 1.2 indicated antagonism. All viability experiments were performed two time (*n* = 2) to ensure reproducibility

### Proliferation analysis

In order to assess proliferation, 2500 cells were seeded in three replicates and followed for 120 h. Cell proliferation was estimated by measuring metabolism using resazurin assay (as described below). Measurements were taken at 5, 24, 48, 72, 96, and 120 h timepoints. Data were normalized against 5 h measurement. Doubling time was computed using Graphpad Prism 8 using exponential curve fit model. To test whether drug inhibition depends on cell proliferation, cell doubling time was plotted against 500 nM JQ1 viability, 400 µM TMZ viability, AUC of JQ1, and AUC of TMZ, followed by a correlation analysis. Proliferation experiments were repeated two times (*n* = 2).

### Cell cycle analysis

To analyze cell cycle, cells were exposed to 500 nM JQ1, 750 nM MLN8237 or 400 µM TMZ (median EC_50_ value for each of the inhibitors) alone or in combination for 72 h. The combination inhibition was as follows: JQ1 + TMZ or JQ1 + MLN8237. Cells were detached and stained using FxCycle PI/RNase Staining Solution (cat# F10797, Thermo Fisher Scientific) according to manufacturer’s protocol. Population of 50 s000 cells was recorded on CytoFlex S (Beckman Coulter). Analysis was performed using CytExpert analysis software (Beckman Coulter). The cell cycle analysis was performed twice (*n* = 2) for JQ1 + TMZ and three times (*n* = 3) for JQ1 + MLN8237

### Protein biochemical analysis

Cells were exposed to 500 nM JQ1, 750 nM MLN8237 or 400 µM TMZ alone or in combination for 72 h before they were collected and lysed using Cell Lysis Buffer (cat# 9803, Cell Signaling Technology). Total 20 µg of protein lysate was loaded onto 4–12% Bis Tris gel (cat# NP0335BOX, Thermo Fisher Scientific) and transferred to iBlot Gel Transfer Stacks (cat# IB301001, Thermo Fisher Scientific). Membranes were blocked with 5% bovine serum albumin and probed against Beta-actin (sc-47778, Santa Cruz Biotech), Cleaved Caspase-3 (9661 S) and Phospho-Histone-3 (9706) from Cell Signaling, and Lamin B1 (ab16048, Abcam). Secondary HRP-conjugated anti-mouse (NXA931) or anti-rabbit (NA934V) were obtained from GE Healthcare. Chemiluminescent signal was developed with SuperSignal West Pico Chemiluminescent Substrate (cat# 34580, Thermo Fisher Scientific) or SuperSignal West Femto Chemiluminescent Substrate (cat# 34095, Thermo Fisher Scientific) and detected on ImageQuant LAS4000 (GE Healthcare). All protein biochemical assays were performed twice (*n* = 2).

### Resazurin assay

To assess cell viability, 1:10 Resazurin reagent (cat# R7017, Sigma) was added to cells and incubated for 4 h at 37 °C. Fluorescence was detected by excitation at 530 nm and emission at 590 nm using Synergy™ HTX multi-mode reader (BioTek).

### RT-qPCR

The whole RNA was isolated with Trizol reagent (cat# 10296010, Thermo Fisher Scientific) and RNeasy Mini Kit (cat# 74104, Qiagen). RNA concentration was measured with Qubit^®^ RNA BR Assay Kit (cat# Q10210, Thermo Fisher Scientific). cDNA was synthesized from total RNA using SuperScript VILO cDNA Synthesis Kit (cat# 11754050, Thermo Fisher Scientific). Expression of mRNA was determined by SYBR Green PCR Master Mix chemistry (cat# 4334973, Thermo Fisher Scientific), using primer pairs as follows: *MYC* (TGCTCCATGAGGAGACAC and GTGATCCAGACTCTGACCTT), *MYCN* (AAGAACCCAGACCTCGAGTTTGAC and GCAGCAGCTCAAACTTCTTCCAGA), *LGR5* (GTTTCCCGCAAGACGTAACT and CAGCGTCTTCACCTCCTACC) and *GAPDH* (CCGACCCCTTCATTGACCTCAACT and ATATTTCTCGTGGTTCACACCCAT). Expression of *GAPDH* was used as endogenous control. The data were analyzed by comparative ΔΔC_t_ method as previously described^51^. Fold-change expression was normalized against immortalized astrocytes. The RT-qPCR experiments were performed in three technical replicates (*n* = 3).

### RNA sequencing

To determine direct JQ1 inhibition response, cells were exposed to JQ1 for 6 h prior collection and extraction of RNA using RNeasy Mini Kit (cat# 74104, Qiagen). RNA-Seq was performed at Uppsala Genome Center, SciLifeLab, Uppsala University on Ion-Proton platform using Ion AmpliSeq Transcriptome Human Gene Expression panel (Thermo Fischer Scientific). RNA sequencing experiments were performed in three technical replicates (*n* = 3). Raw data from the expression analysis are provided in GEO accession number GSE138942.

### Patient data

Expression data for all patient-derived glioblastoma cell lines was downloaded from HGCC resource (http://hgcc.se/) and analyzed using GraphPad Prism 8. All information was coded and no personal information about patients was presented at any point. Expression of *AURKA* and *MYCN* was downloaded from R2 database (https://hgserver1.amc.nl/cgi-bin/r2/main.cgi) and analyzed using GraphPad Prism 8.

### Differential expression analyses

Differential gene expression analyses were performed in R. Specifically, in order to test for significant transcriptional differences between matched DMSO treated and JQ1 samples we conducted a paired t-test using the *t.test* function and corrected the obtained p-values for multiple testing using the *qvalue* function. For each comparison, the top 100 up- and down-regulated genes, as measured by the *q*-value, were retained for further analysis.

### Gene set enrichment and gene set overlap analyses

Gene set enrichment analyses (GSEAs) were conducted using the GSEA module on the genepattern web platform. Statistics were calculated using gene set permutations. Unbiased analyses were performed against five databases of gene sets (Hallmark, curated gene sets (C2), Transcription factor targets (TFT), Gene Ontology (C5) and Oncogenic Signatures (C6)). GSEA was performed on microarray data available on www.hgcc.se and published in ref. ^19^. Top 50 genes upregulated in JQ1-sensitive and JQ1-resistant cell lines was used to define the sensitivity signature (sensitivity gene sets).

Gene set overlap analyses were conducted via the GSEA homepage (http://software.broadinstitute.org/gsea/). Specifically, the top 100 up- or down-regulated genes were compared against gene sets from five different databases: Hallmarks (H), Curated gene sets (C2), Transcription factor targets (TFT), Gene ontology (C5), Oncogenic signatures (C6).

### Sensitivity signature enrichment in TCGA dataset

In order to map our sensitive-vs-resistant gene signature to GBM patients, we first downloaded a publicly available TCGA gene expression data set together with clinical data set for 539 GBM samples from the UCSC cancer browser. The included gene expression values were measured on the AffyU133a microarray platform, RMA normalized and log2 transformed by the Broad institute of MIT. From this initial data, we removed 97 samples that were classified as belonging to the Neural subgroup, producing a final expression array of 442 samples (158 Mesenchymal, 145 Classical, 139 Proneural).

From the initial list of 100 genes used to distinguish between sensitive and resistant cells, 79 were also directly annotated in the TCGA data. Using these 79 genes, we encoded a sensitive-vs-resistant signature by letting the genes upregulated in the sensitive group of cells (*n* = 41) take a value of 1, while the genes upregulated in the resistant group of cells (*n* = 38) were assigned a value of −1. Subsequently, we computed for each of those genes the corresponding expression z-score across all samples in the TCGA data. Finally, we computed pairwise Pearson’s correlation coefficients between the z-score profile (across those 79 genes) from a TCGA sample and the 1/−1 sensitive-vs-resistant signature, such that samples with a strong correlation coefficient would be classified as displaying a gene expression profile similar to the sensitive cell group, while samples with a strong negative correlation coefficient would be considered to exhibit an expression profile more similar to the resistant cell group.

For survival analyses between samples with a more sensitive or resistant expression profile, respectively, we then identified for every subgroup the samples with a Pearson’s correlation coefficient >= 0.2 or <= −0.2, respectively. Due to the close association of subgroups with either the sensitive or resistant profile, the results were highly skewed with 79 sensitive vs 7 resistant samples for the Proneural subgroup, 2 sensitive vs 107 resistant samples for the Mesenchymal subgroup, and 55 sensitive vs 12 resistant samples for the Classical subgroup. Given the low number of sensitive Mesenchymal samples, we thus only performed overall survival comparisons between sensitive and resistant samples within the Classical and Proneural subgroups, respectively. Survival analyses were performed in R using the survival (v2.41-3) package.

### Statistical analysis

Dose–response curves and EC_50_ values were calculated using GraphPad Prism 8. EC_50_ values were computed for each inhibitor and given cell line using non-linear regression model (four parameters). Combination indices were computed using CompuSyn^50^. Student’s *t*-test was applied to calculate significant difference after inhibition. Significant difference in cell viability was computed using one-way analysis of variance (ANOVA) with Tukey’s correction for multiple comparisons. Data collected follow normal distribution with a similar variance between two groups being compared. Error bars represent mean value with standard error of mean. Sample size and replicates are indicated per each method described above. All graphs indicating multiple repeated measurements are presented as mean values with standard deviation of mean.

## Supplementary information

Supplementary Figure and Table Legends

Figure S1

Figure S2

Table S1

Table S2

Table S3

Table S4

Table S5

Table S6

Table S7

Table S8

Table S9

Table S10
