# Supplementary material for: BET and Aurora Kinase A inhibitors synergize against MYCN-positive human glioblastoma cells
Source: Cell Death Dis. 2019 Nov 21;10(12):881. doi: 10.1038/s41419-019-2120-1 (PMC6872649; doi:10.1038/s41419-019-2120-1)
Supplement: Supplementary file 1 — Supplementary Figure and Table Legends [file 41419_2019_2120_MOESM1_ESM.docx]

**Supplementary Figure Legends**

**Figure S1.** Sensitivity subgrouping based on viability at 500nM JQ1 (EC_50_) correlates with subgrouping based on AUC analysis (A). Cell lines showing disagreement in subgrouping using two different methods are indicated. Correlation between cell doubling time and viability at 500nM JQ1 (B), AUC for JQ1 dose-response (C), viability at 400μM TMZ (D), and AUC for TMZ dose response (E). All correlation analyses are performed using Pearson correlation.

**Figure S2.** Survival curves of proneural (PN) GBM patients carrying JQ1-sensitive (red) or JQ1-resistant (blue) signature (A). Patients having JQ1-sensitive signature demonstrate longer median survival (316 days) compared to patients having JQ1-resistant signature (183 days). PN patients’ IDH1 mutation status does not correlate with the JQ1 sensitivity signature (B).

**Supplementary Table Legends**

**Table S1.** Curve fit parameters for EC50 computation

**Table S2.** Differentially expressed genes in JQ1-sensitive and JQ1-resistant cell lines (related to Figure 2A)

**Table S3**. Top 100 genes downregulated in sensitive lines upon JQ1 inhibition

**Table S4**. Top 100 genes upregulated in sensitive lines upon JQ1 inhibition

**Table S5**. Top 100 genes downregulated in resistant lines upon JQ1 inhibition

**Table S6**. Top 100 genes upregulated in resistant lines upon JQ1 inhibition

**Table S7.** Detailed multiple comparison statistical analysis related to Figure 4E

**Table S8.** Detailed multiple comparison statistical analysis related to Figure 4F

**Table S9.** Detailed multiple comparison statistical analysis related to Figure 5C

**Table S10.** Adjusted p-values for multiple comparison of cell cycle analysis (related to Figure 5)
