# Supplementary figures and images for: BET and Aurora Kinase A inhibitors synergize against MYCN-positive human glioblastoma cells
Source: Cell Death Dis. 2019 Nov 21;10(12):881. doi: 10.1038/s41419-019-2120-1 (PMC6872649; doi:10.1038/s41419-019-2120-1)

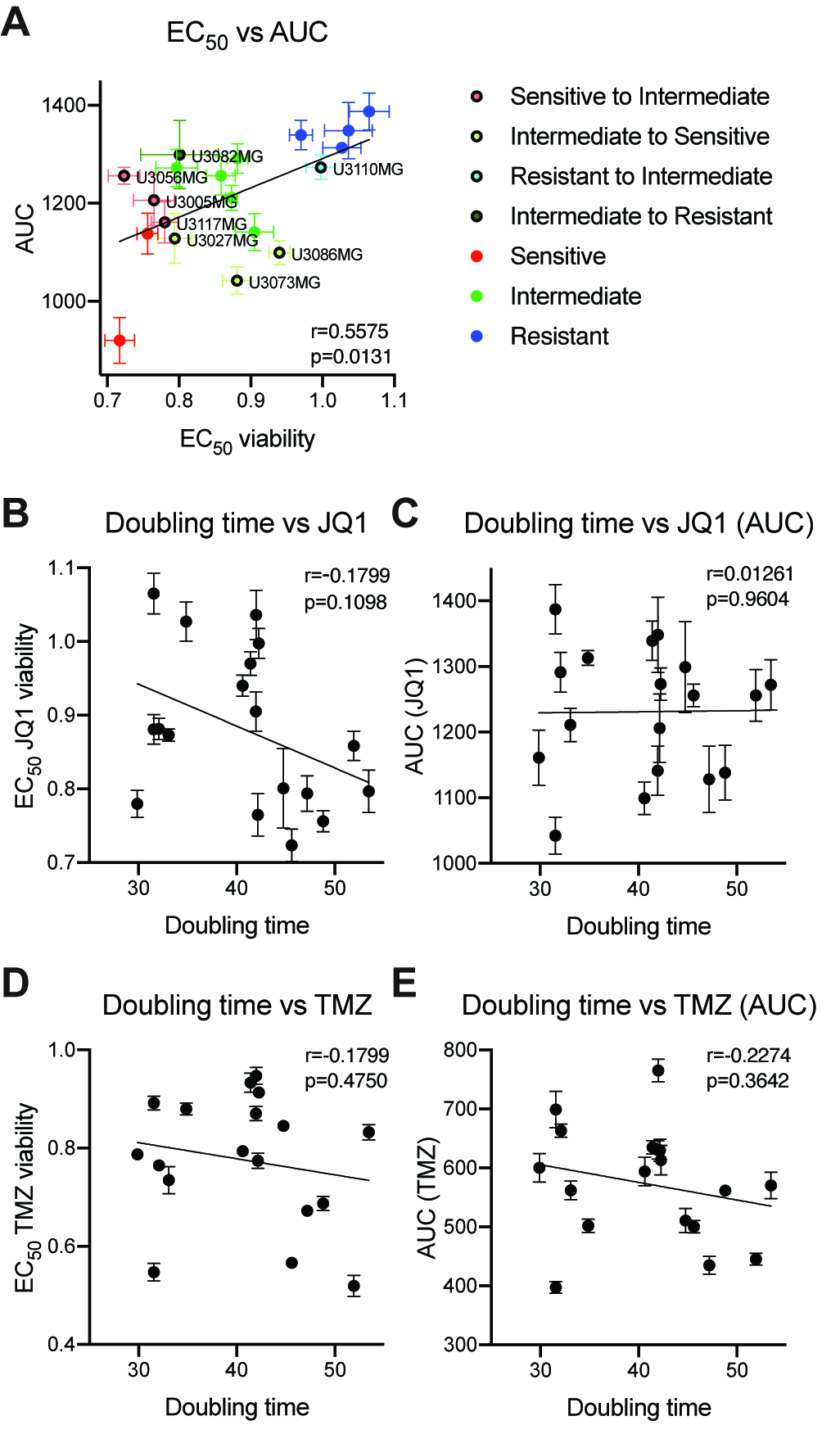

Supplement: Supplementary file 2 — Figure S1 [file 41419_2019_2120_MOESM2_ESM.tif]

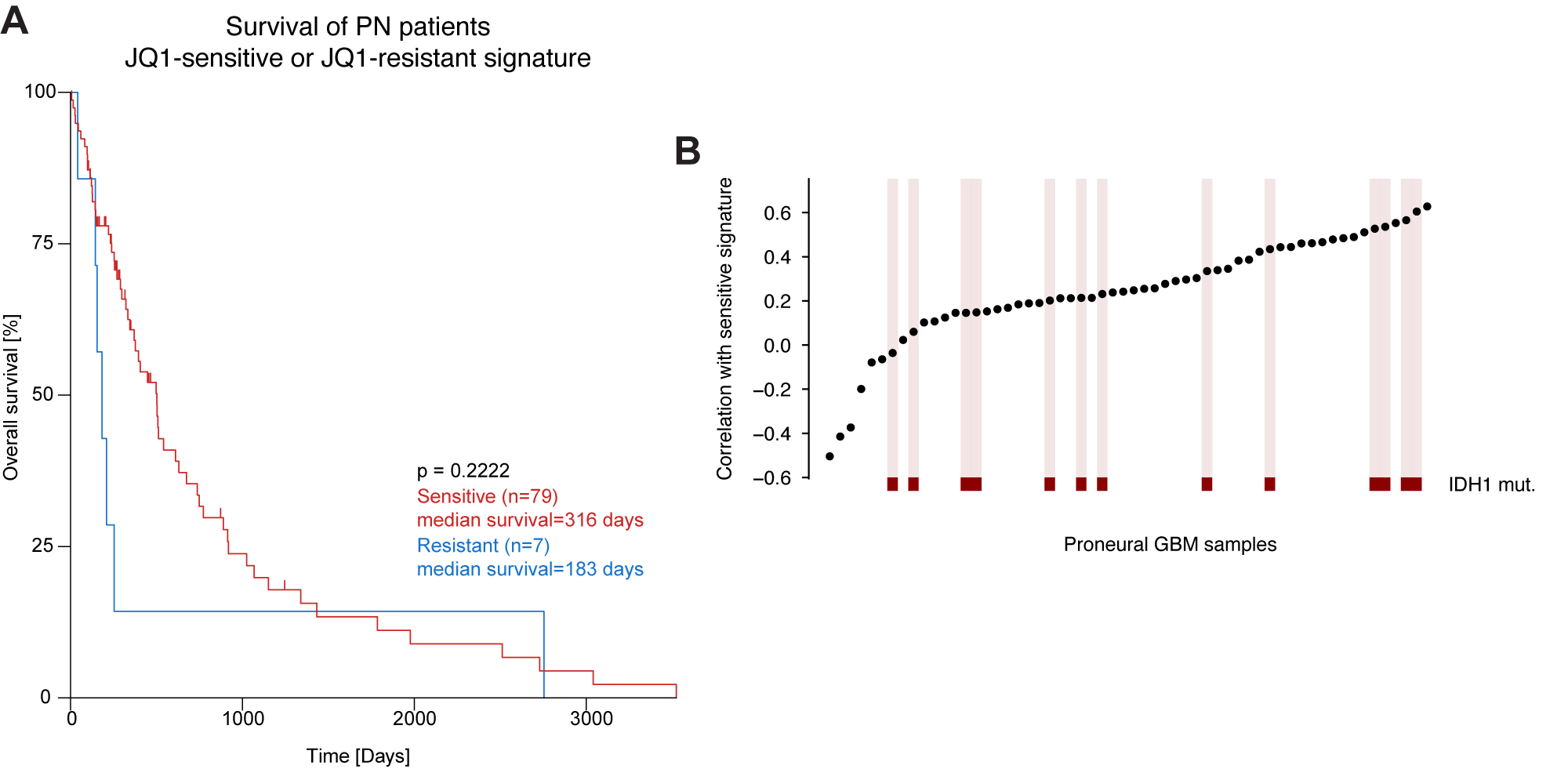

Supplement: Supplementary file 3 — Figure S2 [file 41419_2019_2120_MOESM3_ESM.tif]
